# Why aggregated data falls short: an exploratory survey study on barriers and facilitators in implementing the stepped care model for mental health in primary care

**DOI:** 10.1186/s12875-026-03238-0

**Published:** 2026-02-28

**Authors:** Anders Brantnell, Svea Fredriksson, Amanda Simonsson, Klas Palm

**Affiliations:** 1 Department of Civil and Industrial Engineering, Industrial Engineering and Management, Uppsala University, Lägerhyddsvägen 1, Ångströmlaboratoriet, Uppsala, 752 37 Sweden; 2 Department of Women’s and Children’s Health, Healthcare Sciences and e-Health, Uppsala University, MTC-huset, Dag Hammarskjölds väg 14B, 1 tr, Uppsala, 752 37 Sweden; 3 Department of Psychology, Clinical Psychology, Uppsala University, von Kramers allé 1A och 1C, Uppsala, 752 37 Sweden

**Keywords:** stepped care model, implementation, barriers, facilitators, primary care, healthcare professionals, mental health

## Abstract

**Background:**

Implementing the stepped care model for mental health in primary care organizations is a complex process influenced by various barriers and facilitators. Identifying these barriers and facilitators at the appropriate level is crucial for developing effective implementation strategies. Traditionally, barriers and facilitators are identified at the aggregate level, which may not be optimal due to potential organizational and professional differences. The objectives of this study were to identify the barriers and facilitators to implementing the stepped care model across three primary care organizations in Sweden and to explore differences in barriers and facilitators at the aggregate, organizational, and professional levels.

**Methods:**

An online self-report survey was conducted between January and May 2023 among healthcare professionals (*n* = 33) in three Swedish primary care organizations implementing the stepped care model for mental health. The survey included two open-ended questions regarding barriers and facilitators. Summative content analysis was used to identify frequent barriers and facilitators at the aggregate, organizational, and professional levels. Given the small sample size and early stage of implementation, the study was designed as an exploratory study.

**Results:**

The findings revealed seven frequent barriers and facilitators to implementation: benefits of the model, information and communication, knowledge of the model, propensity to change, resources, support from management and time to work with implementation. At the aggregate level, two barriers (e.g., time to work on implementation) and five facilitators (e.g., resources) were prevalent. At the organizational level, some frequent barriers and facilitators corresponded with those identified at the aggregate level, but there were notable differences between the three organizations. At the professional level, some similarities were observed among psychologists, but no consistent trends were found across other professions.

**Conclusions:**

The study provides three novel contributions to stepped-care implementation research. First, organizational-level analysis reveals context-specific barriers and facilitators that aggregated data overlook, making the organizational level the most informative for guiding implementation. Second, the time required to work on implementation emerges as a distinct barrier. Third, resources function not only as barriers when lacking but also as facilitators when available.

**Supplementary Information:**

The online version contains supplementary material available at 10.1186/s12875-026-03238-0.

## Introduction

Mental health disorders, including depression and anxiety, pose a significant global public health challenge [1, 2]. In the EU alone, approximately 165 million individuals experience mental health and substance use disorders annually, with over half the population in middle- and high-income countries likely to face a mental disorder in their lifetime [3]. The economic burden of mental health disorders is enormous. In 2010, global costs were estimated at US$2.5 trillion, with the EU accounting for €798 billion, and these figures are projected to double by 2030 [3]. By 2022, the WHO reported 508 million women and 462 million men globally suffering from mental health conditions [4]. Given the scale of the issue, improving mental health care is a priority for many governments and health systems. In Europe, including Sweden, primary care often serves as the foundation for mental health services [5, 6]. However, primary care systems face increasing demand, psychologist shortages, and challenges related to aging populations [7–9].

A promising approach to addressing these challenges is the stepped care model [10–12], which aims to provide accessible and effective mental health services [13]. This model begins with the least intensive treatment and adjusts care based on patient progress [14]. Sweden has embraced this model as part of its “Good and Close Care” reform, which seeks to make primary care more patient-centered [15]. However, implementing such a complex model demands consideration of individual, organizational, and systemic factors [16].

Studies on the implementation of stepped care have identified both common and context-specific challenges, which vary depending on the implementation phase, target population, and setting [17–21]. One study examined a depression-focused model across eight regions [18], while another explored its application for personality disorders at two sites [20]. Several studies also assessed the feasibility of the model, including one focused on cancer patients with insomnia across five hospitals [21], and another that investigated its use in district-wide addiction and mental health services [19].

Common barriers to stepped care implementation include resource limitations, interorganizational disagreements, resistance to change, patient motivation and poor integration into practice [17–21]. Many of these barriers are well documented in general implementation research [22], whereas others, such as high rates of comorbid mental health conditions [21] and psychologist shortages [17], are more specific to mental health service delivery. Facilitators to stepped care implementation commonly include clinician training, management support, improved communication, clinical champions, clinician motivation, and organizational readiness [17–21]. While these facilitators are also frequently reported in broader implementation research [22], their relevance is particularly pronounced in mental health settings due to the multidisciplinary nature of care and the reliance on therapeutic engagement [19–21].

Based on implementation research in general, identifying barriers and facilitators is critical for developing effective implementation strategies [23]. Existing implementation studies indicate that the perspectives of various professional groups are particularly important, as different professions may perceive barriers and facilitators differently [24]. Moreover, implementation studies have shown that organizational level factors, such as organization readiness, also influence implementation [22]. However, most existing studies on stepped care implementation focus on aggregated barriers and facilitators across multiple organizations, with only one [20] comparing differences between organizations with varying success rates. None have directly compared professional perspectives on these challenges. Existing methods, in general in implementation research, to identify barriers and facilitators include systematic reviews, interviews, focus groups, frameworks, and observations [22, 25–29]. However, several studies, on stepped care implementation, overlook professional or organizational differences, focusing instead on aggregated barriers and facilitators, and consequently propose implementation strategies based on this aggregated analysis [17–19, 21].

This study presents findings from the early implementation of a stepped care model for mental health in Swedish primary care, focusing on three organizations. In Sweden, primary care is regionally governed and typically delivered through multidisciplinary teams that manage mild to moderate mental health conditions. As part of a regional initiative to introduce stepped care, three primary care organizations were selected by the region to pilot the model. A more detailed description of the Swedish primary care context and the pilot organizations is provided in the Methods section (Setting).

The objectives of this study were to identify the barriers and facilitators to implementing the stepped care model across three primary care organizations in Sweden and to explore differences in barriers and facilitators at the aggregate, organizational, and professional levels. Given the small sample size and early stage of implementation, the study was designed as an exploratory study. Three research questions were posed: What are the barriers and facilitators influencing the implementation of a stepped care model for mental health?; How do these barriers and facilitators differ across aggregated, organization, and profession levels?; At which level should these barriers and facilitators be identified to develop effective implementation strategies?

## Methods

### Study design

An online exploratory self-report survey was conducted between January and May 2023 among healthcare professionals from three Swedish primary care organizations implementing the stepped care model for mental health. To improve the response rate, participants were also given the option to complete the survey offline (i.e., using paper and pen) during a site visit. The survey focused on the ongoing implementation of the stepped care model and included two open-ended questions regarding barriers and facilitators to its implementation. This paper reports the findings from the open-ended questions, while results from the rest of the survey are presented elsewhere.

### Setting

In 2016, there were approximately 1,150 primary care practices in Sweden [30]. This figure still serves as a rough estimate of the current number, as some practices have closed due to bankruptcy, while others have been newly established. Sweden is divided into 21 regions, each responsible for funding and managing its primary care organizations. A typical primary care organization includes a multidisciplinary team of medical doctors (i.e., general practitioners), nurses, physiotherapists, and psychologists who collaborate to provide care to a wide range of patients. Staffing levels within these organizations are proportional to their patient populations, which range from around 3,000 to 30,000 per organization, on average, approximately 8,000–8,500 patients listed. As a result, some organizations serve smaller patient groups, while others manage significantly larger caseloads [30]. Both private and public primary care organizations operate under similar conditions, receiving financial support from the regions. These shared conditions include staff expertise, funding, adherence to national care guidelines, operational hours, and patient accessibility. Adult patients pay an out-of-pocket fee of approximately €25 for primary care services, such as medical consultations or psychologist visits. Primary care manages mild to moderate cases of common mental disorders, such as depression and anxiety, while more complex cases are referred to specialized care.

In Sweden, primary care professionals have varying lengths of formal training. District nurses complete a three-year Bachelor of Science in Nursing followed by a one-year specialist program in primary health care. Psychologists undergo a five-year professional degree program that includes clinical training. Medical doctors complete a six-year medical degree followed by a minimum five-year residency in family medicine. Counselors typically hold a three-year Bachelor of Science in Social Work, often supplemented by clinical postgraduate training.

In 2021, a project was launched to implement the stepped care model for mental health and adapt it to the local context in a Swedish region. Three primary care organizations were selected as the initial group to pilot the model, making them the only viable locations in the region for conducting this study. Each organization appointed a clinical staff member to oversee its implementation, with these roles accounting for 20–30% of a full-time position, depending on the organization. These staff members collaborated with a regional project group responsible for disseminating, developing, and introducing the model’s three core components: assessment, treatment, and follow-up. During the project’s initial years, significant emphasis was placed on establishing routines for patient assessment. In parallel, the heads of the primary care organizations participated in a managerial network that approached the implementation process from a strategic perspective. This dual approach aimed to co-develop the stepped care model with the three pilot organizations while simultaneously implementing it at these sites.

The implementation of the stepped care model involves all professions within primary care organizations, but four are central to its success: nurses, counselors, psychologists, and medical doctors. Nurses serve as the primary point of contact in primary care, often being the first healthcare professionals to assess and interact with patients. The care process typically begins with nurses triaging patients to the most appropriate profession for further assessment. This initial assessment is frequently conducted by counselors or psychologists. Medical doctors become involved when questions arise regarding medication, sick leave, or somatic symptoms.

Counselors are essential in supporting patients with emotional and psychological challenges. Their mental health expertise helps identify early signs of psychological distress and guides patients through less intensive interventions, aligning with the model’s principle of using the least intrusive approach first. Psychologists contribute specialized expertise in assessing mental health issues and providing therapeutic interventions, particularly for more complex cases. In the stepped care model, psychologists play a pivotal role in treating moderate psychological problems. Medical doctors are integral to the model, focusing on assessments, managing comorbid conditions, and prescribing medication. Across all professions, collaboration ensures a holistic and integrated approach to patient care.

Following the pilot phase, full-scale implementation of the stepped care model is planned for 2025, with the model being extended to all primary care organizations in the region, approximately 50 in total.

### Data collection

To collect comprehensive data on the barriers and facilitators to implementing the stepped care model for mental health, a survey was created and distributed using SurveyMonkey. The heads of all primary care organizations were asked to share the survey link, with three reminders sent to encourage participation. In addition, the individuals responsible for overseeing the model’s implementation within each organization were tasked with distributing the survey to all employees. To further boost participation, the regional-level coordinator for the stepped care model directly engaged with the organization heads to emphasize the importance of completing the survey.

To improve the response rate, all organizations were offered the option to complete the survey offline using paper and pen. Following this, a researcher visited two of the organizations to facilitate participation. The third organization was unable to arrange a physical meeting; however, given the low response rate, visiting two organizations was considered beneficial. During the visits, the researcher attended staff workplace meetings, explained the study, presented the survey, and provided staff with the opportunity to complete it manually. Completed paper surveys were later manually entered into SurveyMonkey.

Conducting this survey posed expected challenges, as the research followed the ongoing implementation of the stepped care model, a process inherently complex and demanding for the participating organizations.

The survey targeted all healthcare professions within the primary care organizations, and as a result, respondents included counselors, nurses, psychologists, medical doctors, biomedical analysts, medical secretaries, and heads of the organizations. All employees in each of the three organizations were invited to participate, regardless of professional role. For this study, we chose to focus on the professions most relevant to the implementation of the stepped care model, namely counselors, nurses, psychologists, and medical doctors (*n* = 33). The survey was distributed to all clinical staff (100%) across the three organizations.

A formal sample size calculation was not conducted, as the study could only include the three primary care units that were involved in the implementation process. Because no additional units were eligible for inclusion, calculating an a priori required sample size (e.g., estimating how many respondents would be needed from each professional group) would not have been meaningful or actionable.

Furthermore, GDPR regulations prevented us from obtaining exact numbers of staff per organization at the time of data collection. However, based on publicly available information from 2023 to 2025 (i.e., vacant job announcements, primary care organizations’ social media pages, and press releases), we were able to estimate that Organization 1 had approximately 19,200 listed patients and 115 employees; Organization 2 had 13,000 listed patients and 50 employees; and Organization 3 had 7,200 listed patients and 30 employees. The specific public sources are not disclosed for anonymity reasons.

Using the number of registered patients, we also estimated the approximate staffing composition (see Additional file 1 for calculation details). These estimations suggest that the three organizations together employed approximately 20 medical doctors, 35 nurses, 7 psychologists, and 4 counselors, amounting to 79 individuals across the relevant staff categories. Based on this estimate, the survey achieved an approximate response rate of 42% (*n* = 33). Details on the respondents and their distribution across organizations are provided in Table 1.

**Table 1:** Study respondents categorized by profession and organization

Respondents (*n*)	Councelors	Nurses	Psychologists	Medical doctors	Total per organization
Organization 1	2	4	2	5	13
Organization 2	1	3	2	2	8
Organization 3	1	6	2	3	12
Total for all organizations	4	13	6	10	33

The table presents the number of respondents and their professions from each of the three organizations studied

### The survey

The survey consisted of 11 questions, two of which were open-ended and form the focus of this study. Additionally, two demographic questions were included: one about the respondent’s organization name and another about their profession. The two open-ended questions on barriers and facilitators were phrased as follows:

- (A) *What are the most important facilitating factors for successfully implementing the stepped care model? Please list up to five factors and provide complete sentences.*
- (B) *What are the most important hindering factors for successfully implementing the stepped care model? Please list up to five factors and provide complete sentences.*

The survey introduction explicitly stated that the focus was on the implementation of the stepped care model for mental health within the respondent’s own organization (see Additional file 2 for an English translation of the survey).

### Data analysis

Given the exploratory nature of this study and the small sample size (*n* = 33), we intentionally limited the quantitative component to descriptive statistics and visual comparison using spider diagrams. These approaches were selected to avoid overinterpreting sparse data and to align with the study’s aim of generating early insights rather than testing hypotheses. More advanced inferential analyses were considered inappropriate due to limited statistical power and the heterogeneity across organizations and professional groups.

Data analysis followed summative content analysis, a method suitable for analyzing open-ended survey data [31]. The process was divided into several steps. Initially, all responses to the two questions on barriers and facilitators were listed in an Excel spreadsheet. Similar answers, or quotes, were then grouped together, and initial labels were assigned to the barriers and facilitators.

To enhance analytic rigor, the first and last authors independently reviewed an initial subset of responses to confirm the consistency of grouping and labeling. Discrepancies in labeling were discussed until agreement was reached, and the resulting shared understanding was used to guide the continued coding of the full dataset. This procedure increased the confirmability of the coding process and reduced the risk of researcher bias [32].

Afterward, final labels were created, and the number of responses supporting each barrier and facilitator was counted. A summary table was then constructed, displaying the three organizations and the number of responses supporting each barrier and facilitator (see Additional file 3 for details). Throughout this process, all authors reviewed preliminary summaries to ensure that labels accurately reflected the underlying data, thereby strengthening credibility [32].

Based on this data, the most frequent barriers and facilitators were identified, and the subsequent analysis focused on these. We used descriptive statistics to present barriers and facilitators at the aggregate and organization levels, while for the professional level, we employed spider diagrams [33, 34] to illustrate the frequency of each barrier and facilitator. Spider diagrams, which are commonly used to analyze and visualize various components within innovation ecosystems, were deemed suitable for illustrating barriers and facilitators in the context of implementing a complex intervention. The spider diagram was created using Microsoft Excel, with web threads and bars added manually during the design process. Additionally, we compared the barriers and facilitators at the professional level within and across organizations to explore whether there were any organizational or professional consensus regarding the factors. Finally, we compared the frequencies of barriers and facilitators at the aggregate, organizational, and professional levels, both within the same organization and across all organizations, in order to identify the most effective level for recognizing barriers and facilitators and, ultimately, for developing implementation strategies.

## Findings

### Barriers and facilitators at the aggregated level

The results identified 24 barriers and facilitators. Interestingly, each barrier corresponds to a corresponding facilitator, and vice versa (see Additional file 3 for a complete list of all barriers, facilitators, and their frequencies). To facilitate comparison between the three organizations and professions, we focused on the seven most frequently reported barriers and facilitators (hereafter referred to as “factors”) (see Table 2 for the frequencies of these seven factors).

**Table 2:** The seven most frequent factors influencing the implementation of the stepped care model

Factors	Barrier, *n* (percentage)	Facilitator, *n* (percentage)
Benefits of the model	5 (15%)	7 (21%)
Information and communication	6 (18%)	9 /27%)
Knowledge of the model	4 (12%)	3 (9%)
Propensity to change	6 (18%)	1 (3%)
Resources	10 (30%)	9 (27%)
Support from management	3 (9%)	7 (21%)
Time to work with implementation	13 (39%)	8 (24%)

The table presents the seven most common barriers and facilitators influencing the implementation of the stepped care model for mental health across the three primary care organizations studied. It also displays the number and percentage of each barrier and facilitator

Table 3 presents quotes illustrating the seven factors. Each factor is represented by one quote for a barrier and one for a facilitator. As shown in Table 2, two barriers are prevalent across the three organizations: time to work on implementation (*n* = 13) and resources (*n* = 10). In contrast, five facilitators are most frequently cited: resources (*n* = 9), information and communication (*n* = 9), time to work on implementation (*n* = 8), support from management (*n* = 7), and the benefits of the model (*n* = 7).

**Table 3:** Quotes illustrating the seven most frequent factors influencing the implementation of the stepped care model

Factors	Quotes from the survey data
Benefits of the model	“That you clearly see the benefits of the model” (facilitator, medical doctor) “That you don’t see any immediate benefit with the approach in your own work or for the patient” (barrier, nurse)
Information and communication	“Continuous information about how it [implementation of the model] is supposed to be done” (facilitator, psychologist) “Inadequate communication with the staff” (barrier, nurse)
Knowledge of the model	“Unclarity on what the stepped care entails” (barrier, medical doctor) “Clarity about what it [the model] means, which steps there are and which patient belongs to which step” (facilitator, medical doctor)
Propensity to change	“Difficult to change practice” (barrier, nurse) “Open for suggestions” (facilitator, nurse)
Resources	“Lack of staff” (barrier, counselor) “That there is an opportunity to collaborate with colleagues regarding assessment” (facilitator, psychologist)
Support from management	“That the manager gives room for implementation” (facilitator, psychologist) “That the manager does not support the introduction” (barrier, counselor)
Time to work with implementation	“Lack of time” (barrier, medical doctor) “Time to learn and familiarize yourself with the material” (facilitator, psychologist)

The table presents example quotes for the seven most frequent factors influencing the implementation of the stepped care model for mental health across the three primary care organizations studied. For each factor, one quote represents a barrier, and another represents a facilitator

### Barriers and facilitators at the organizational level

When focusing on the organizational level, a more nuanced picture emerges regarding barriers and facilitators. The 13 respondents from Organization 1 mentioned all seven barriers and facilitators, with two barriers—resources (*n* = 6) and time to work with implementation (*n* = 5)—and two facilitators—information and communication (*n* = 7) and resources (*n* = 6)—standing out. In Organization 2, the eight respondents mentioned six of the seven barriers and facilitators, with one barrier—propensity to change (*n* = 3)—being particularly prominent. No facilitators were mentioned by multiple respondents in this organization. In Organization 3, the eight respondents mentioned all seven barriers and facilitators, with two barriers—time to work with implementation (*n* = 6) and information and communication (*n* = 3)—and three facilitators—time to work with implementation (*n* = 3), support from management (*n* = 3), and benefits of the model (*n* = 3)—being the most frequently cited (for details, see Additional file 3).

To summarize, there are notable differences and similarities between the three organizations in terms of barriers and facilitators to implementation. In terms of similarities, time to work with implementation is among the most frequent barriers in both Organization 1 and Organization 3. Regarding differences, several barriers and facilitators were mentioned by only one organization: resources (Organization 1, barrier), information and communication (Organization 1, facilitator), resources (Organization 1, facilitator), propensity to change (Organization 2, barrier), information and communication (Organization 3, barrier), time to work with implementation (Organization 3, barrier), support from management (Organization 3, barrier), and benefits of the model (Organization 3, barrier).

### Barriers and facilitators at the professional level

**Fig. 1:**
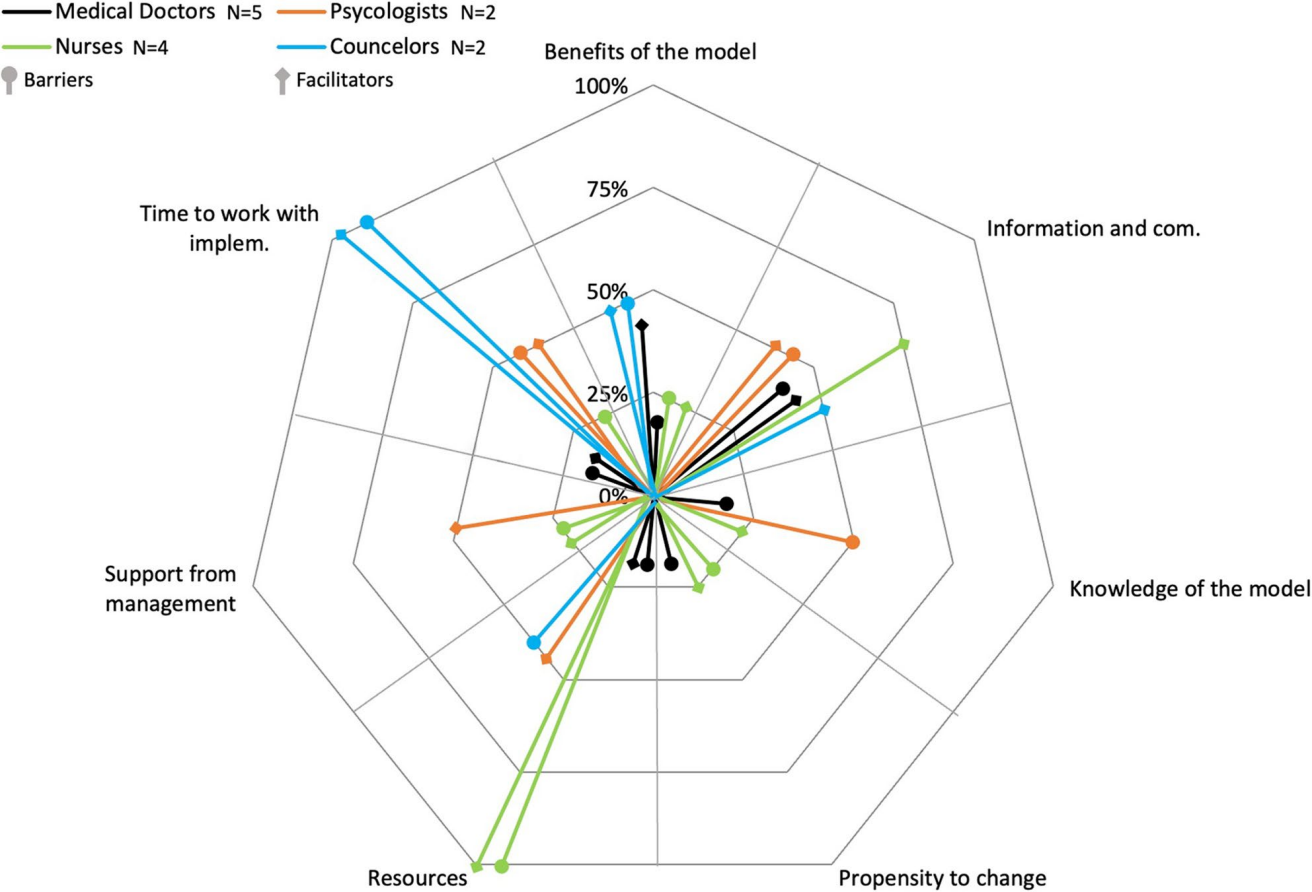
Barriers and facilitators to implementing the stepped care model for mental health in Organization 1. Legend: The figure illustrates the barriers and facilitators to implementing the stepped care model for mental health in Organization 1. It displays the percentage of respondents in each of the four professional categories for the seven identified barriers and facilitators

When focusing on the professions at individual organizations, the similarities and differences in terms of barriers and facilitators become even more apparent. In Organization 1, among medical doctors, the most frequently cited barrier is information and communication (*n* = 2, 20%), though it is still mentioned by only a few. In total, medical doctors mentioned six barriers. This suggests that there is no organizational consensus among medical doctors regarding the barriers in Organization 1. The most frequent facilitators are information and communication and the benefits of the model (*n* = 2, 20%). However, in total, medical doctors mentioned only three facilitators. Again, there is no organizational consensus among medical doctors on facilitators in Organization 1 (for details, see Fig. 1).

**Fig. 2:**
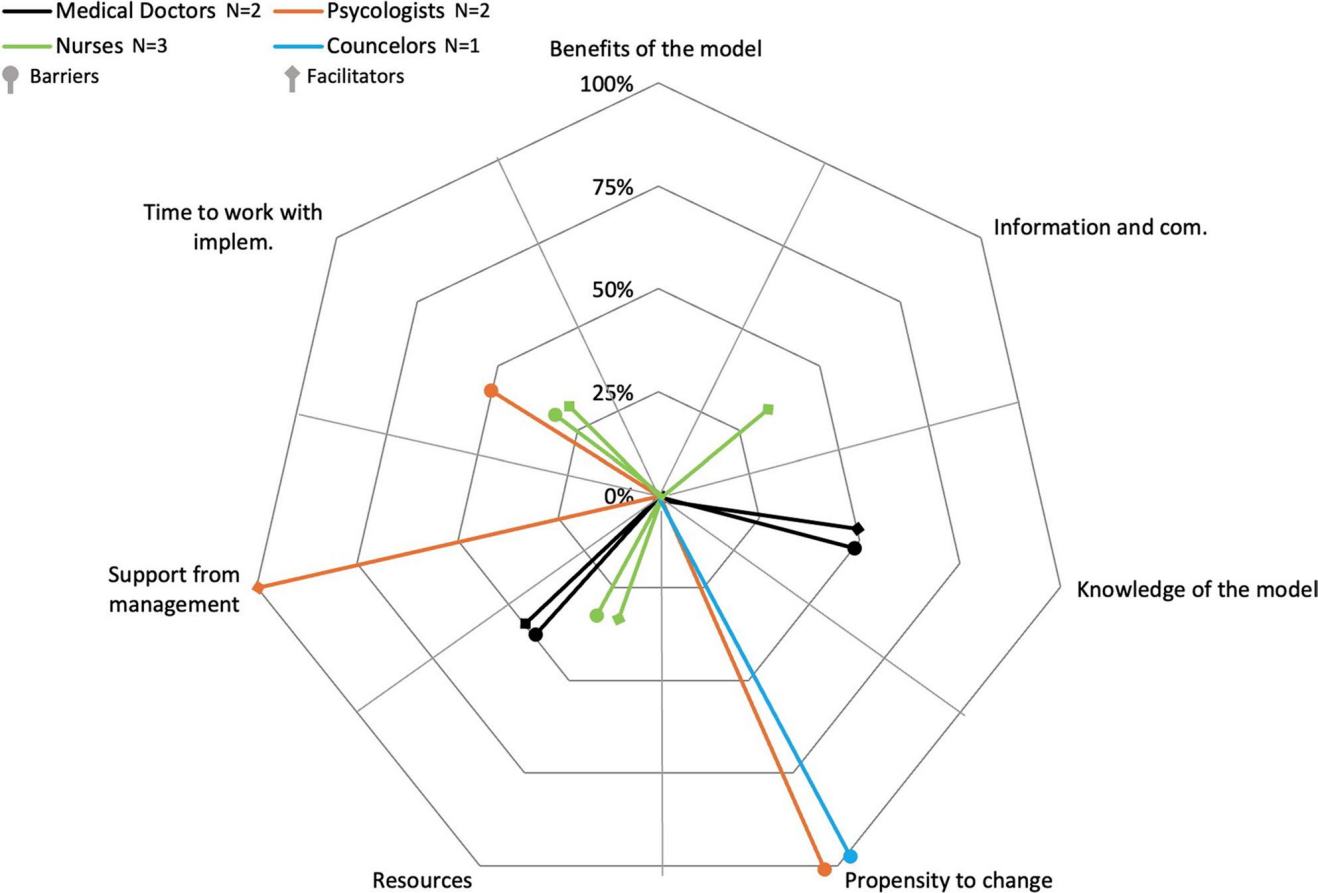
Barriers and facilitators to implementing the stepped care model for mental health in Organization 2. Legend: The figure illustrates the barriers and facilitators to implementing the stepped care model for mental health in Organization 2. It displays the percentage of respondents in each of the four professional categories for the seven identified barriers and facilitators

In Organization 2, among medical doctors, there are no prevalent barriers or facilitators. Instead, all barriers and facilitators are mentioned by only one medical doctor (*n* = 1, 50%). In total, two barriers and two facilitators are cited: knowledge of the model and resources. Therefore, there is no organizational consensus among medical doctors on the barriers and facilitators in Organization 2 (for details, see Fig. 2).

**Fig. 3:**
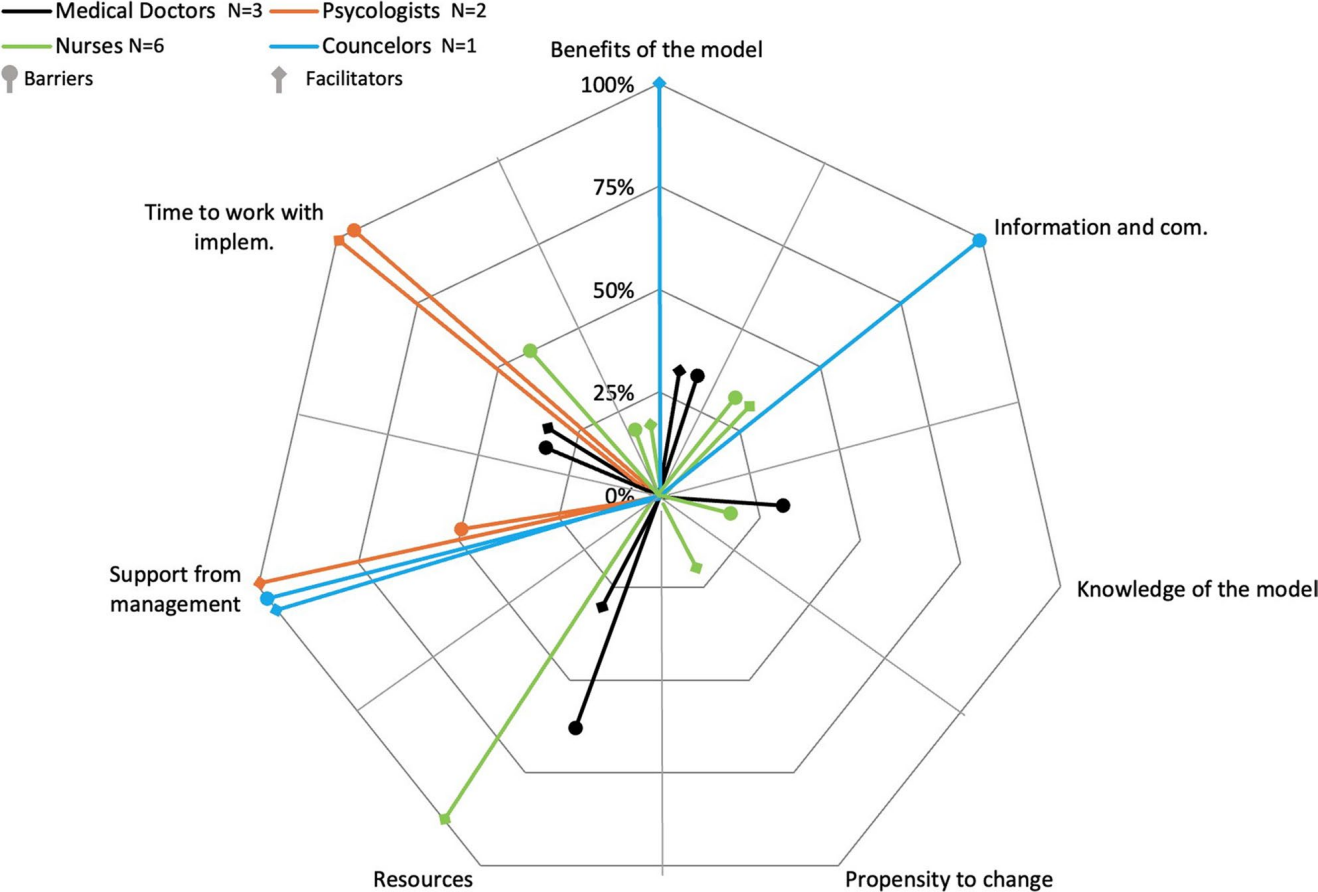
Barriers and facilitators to implementing the stepped care model for mental health in Organization 3. Legend: The figure illustrates the barriers and facilitators to implementing the stepped care model for mental health in Organization 3. It displays the percentage of respondents in each of the four professional categories for the seven identified barriers and facilitators

In Organization 3, among medical doctors, the most frequently mentioned barrier is resources (*n* = 2, 66%), cited by the majority. In total, four barriers are mentioned by medical doctors. However, there is no clear organizational consensus on barriers in Organization 3. No frequent facilitators are reported. In total, medical doctors mention three facilitators, and once again, there is no organizational consensus on facilitators in Organization 3 (for details, see Fig. 3).

In Organization 1, among nurses, the most frequent barrier is resources (*n* = 4, 100%), mentioned by all nurses. In total, five barriers are mentioned by nurses. However, there is no clear organizational consensus among nurses on barriers in organization 1. The most frequent facilitators are resources and information and communication (*n* = 4, 100%), both mentioned by all nurses. In total, six facilitators are mentioned by nurses, but again, there is no clear organizational consensus among nurses on facilitators in Organization 1 (for details, see Fig. 1). In Organization 2, among nurses, there are no frequent barriers or facilitators, as all barriers and facilitators are mentioned by only one nurse (*n* = 1, 33%). In total, two barriers and three facilitators are mentioned. Therefore, there is no organizational consensus among nurses on barriers and facilitators in Organization 2 (for details, see Fig. 2). In Organization 3, among nurses, the most frequent barrier is time to work with implementation (*n* = 3, 50%), mentioned by 50% of the nurses. In total, four barriers are mentioned by nurses. Once again, there is no organizational consensus among nurses on barriers in organization 3. Resources is the most frequent facilitator (*n* = 6, 100%), mentioned by all nurses. In total, four facilitators are mentioned by nurses, but once again, there is no clear organizational consensus on facilitators in Organization 3 (for details, see Fig. 3).

In Organization 1, among psychologists, there are no frequent barriers or facilitators, as all barriers and facilitators are mentioned by only one psychologist (*n* = 1, 50%). In total, three barriers and four facilitators are mentioned by psychologists. Therefore, there is no organizational consensus among psychologists on barriers and facilitators in Organization 1 (for details, see Fig. 1). In Organization 2, among psychologists, the most frequent barrier is propensity to change (*n* = 2, 100%), mentioned by all psychologists. Additionally, time to work with implementation is mentioned as a barrier, indicating near organizational consensus on barriers in Organization 2. The most frequent and only facilitator is support from management (*n* = 2, 100%), mentioned by all psychologists. In conclusion, there is organizational consensus on facilitators among psychologists in Organization 2 (for details, see Fig. 2). In Organization 3, among psychologists, the most frequent barrier is time to work with implementation (*n* = 2, 100%), mentioned by all psychologists. Additionally, support from management is mentioned as a barrier, indicating near organizational consensus on barriers in Organization 3. The most frequent and only facilitators are time to work with implementation and support from management (*n* = 2, 100%), mentioned by all psychologists. In conclusion, there is organizational consensus on facilitators among psychologists in Organization 3 (for details, see Fig. 3).

In Organization 1, among counselors, the most frequent barrier and the most frequent facilitator is time to work with implementation (*n* = 2, 100%), mentioned by all counselors. In total, counselors mention three barriers and three facilitators. However, there is no clear organizational consensus on barriers and facilitators in Organization 1 (for details, see Fig. 1). In Organization 2, among counselors, the only barrier mentioned is propensity to change (*n* = 1, 100%). No facilitators are mentioned. Since there is only one counselor in Organization 2, no conclusions can be drawn on similarities or differences between counselors in this organization (for details, see Fig. 2). In Organization 3, among counselors, two barriers and two facilitators are mentioned (*n* = 1, 100%), but as there is only one counselor in Organization 3, no conclusions can be drawn on similarities or differences between counselors in this organization (for details, see Fig. 3).

Barriers and facilitators at the professional level across all organizations.

In organizations 1, 2, and 3, there are no common frequent barriers or facilitators among medical doctors, resulting in no professional consensus on barriers and facilitators. Similarly, there are no common frequent barriers among nurses across the three organizations, indicating no professional consensus on barriers. However, in organizations 1 and 3, resources is the most common facilitator mentioned by nurses. Additionally, in Organization 1, information and communication is also mentioned as a frequent facilitator, but this variation prevents a clear professional consensus on facilitators among nurses. Among psychologists, there are no common frequent barriers in organizations 1, 2, and 3, so no professional consensus is found on barriers. However, in organizations 2 and 3, support from management is the most common facilitator. In Organization 3, time to work with implementation is also mentioned as a frequent facilitator, indicating that there is no clear professional consensus on facilitators. For counselors, there are no common frequent barriers or facilitators across organizations. However, both organizations 1 and 3 mention benefits of the model as a facilitator, but this is not enough for a clear professional consensus on barriers and facilitators (for details, see Figs. 1, 2 and 3).

Table 4 summarizes the findings concerning professional-level barriers and facilitators both within and between the three organizations. The table shows that there is organizational consensus on facilitators in organizations 2 and 3 concerning psychologists. Additionally, there is near organizational consensus on barriers in organizations 2 and 3 related to psychologists. However, in the majority of cases (24 out of 28 possible), there is either “no consensus” or “no clear consensus” regarding barriers and facilitators at the organizational and professional levels.

**Table 4:** Distribution of professional and organizational consensus on barriers and facilitators to implementing the stepped care model

Professions	Medical doctors	Nurses	Psychologists	Counselors
Organization 1	B^1^: no org^2^ consensus F^3^: no org consensus	B: no clear org consensus F: no clear org consensus	B&F: no org consensus	B&F: no clear org consensus
Organization 2	B&F^4^: no org consensus	B&F: no org consensus	B: *near to org consensus* F: *org consensus*	N/A
Organization 3	B: no clear org consensus F: no org consensus	B: no org consensus F: no clear org consensus	B: *near to org consensus* F: *org consensus*	N/A
Organizations 1–3	B&F: no prof^5^ consensus	B: no prof consensus F: no clear prof consensus	B: no prof consensus F: no clear prof consensus	B&F: no prof consensus

^1^ B stands for barrier^2^ Org stands for organization^3^ F stands for facilitator^4^ B&F stands for barriers and facilitators^5^ Prof stands for professional

The table presents the distribution of professional consensus (i.e., the level of agreement on barriers and facilitators within each profession) across the four professions in individual organizations. It also shows the distribution of organizational consensus (i.e., the level of agreement on barriers and facilitators across all three organizations) for the four professions. Both professional and organizational consensus fall into one of four categories: no consensus, no clear consensus, near consensus, or consensus

### Comparing different levels to capture the barriers and facilitators

Across all organizations, two barriers and five facilitators were identified as prevalent. To prioritize, managers would likely focus on two key barriers: time to work with implementation (*n* = 13) and resources (*n* = 10), and two key facilitators: resources (*n* = 9) and information and communication (*n* = 9). However, when considering individual organizations, the recommendations differ. In Organization 1, managers would prioritize two barriers: resources (*n* = 6) and time to work with implementation (*n* = 5), and two facilitators: information and communication (*n* = 7) and resources (*n* = 6). These recommendations align closely with those for the aggregated level. In Organization 2, the recommendation would be to focus on the barrier of propensity to change (*n* = 3), which deviates from the broader organizational-level recommendations. In Organization 3, the focus would be on two barriers: time to work with implementation (*n* = 6) and information and communication (*n* = 3), as well as three facilitators: time to work with implementation (*n* = 3), support from management (*n* = 3), and benefits of the model (*n* = 3). These recommendations are less aligned with the overall findings across organizations (Table 5).

**Table 5:** Cross-organizational comparison

Organization 1 (n = 13)	Resources as a barrier: 6/13 = 46%
	Time as a barrier: 5/13 = 38%
	Information and communication as a facilitator: 7/13 = 54%
	Resources as a facilitator: 6/13 = 46%
Organization 2 (n = 8)	Propensity to change as a barrier: 3/8 = 38%
	No facilitator mentioned by more than one respondent.
Organization 3 (n = 12)	Time as a barrier: 6/12 = 50%
	Information and communication as a barrier: 3/12 = 25%
	Time as a facilitator: 3/12 = 25%
	Support from managemement as a facilitator: 3/12 = 25%
	Benefits of the model as a facilitator: 3/12 = 25%

The table presents a cross-organizational comparison of the most frequently reported barriers and facilitators in the three primary care organizations, described through frequencies and corresponding percentages

At the professional level, the recommendations for organizations 2 and 3 would focus on the facilitators, as there is organizational consensus among psychologists in these organizations regarding the key facilitators. Additionally, there is near consensus on the barriers among psychologists in both organizations. However, for the other professions, no consensus or clear consensus was identified. This indicates that professionals within the same organization, as well as across organizations, did not consistently agree on the barriers and facilitators to implementation.

## Discussion

This exploratory survey study presents findings from the early implementation of a stepped care model for mental health in Swedish primary care, focusing on three organizations. Our findings contribute to understanding the barriers and facilitators to implementation and shed light on the complexities of introducing stepped care interventions in mental health. Importantly, we also clarify which findings appear endemic to primary-care change processes in general and which are more specific to the stepped care model.

The first research question was: “What are the barriers and facilitators influencing the implementation of a stepped care model for mental health?” Our findings identified seven prominent barriers and facilitators, six of which align with previous research on stepped care implementation: (1) benefits of the model [19], (2) information and communication [17, 18, 20], (3) knowledge of the model [17, 20, 21], (4) propensity to change [19, 21], (5) resources [17–19, 21], and (6) support from management [17, 20]. These factors are also commonly reported in generic primary care implementation research [35–37], suggesting that they reflect broader primary-care change processes rather than features unique to stepped care.

In our study, the most prominent barrier was the time required to work on implementation, a factor not identified in existing research on stepped care implementation. While “time” could be seen as part of “resources”, a commonly cited barrier in previous studies on stepped care implementation [17–19, 21], our findings distinguished it as a critical factor in its own right. This represents a novel contribution to stepped-care literature. The concept of readiness for implementation, as described in implementation research and in the widely used Consolidated Framework for Implementation Research (CFIR) [22], may also relate to time. Readiness is defined as “tangible and immediate indicators of organizational commitment to its decision to implement an intervention” [22], and sufficient time is arguably one such indicator.

Importantly, time constraints have also been identified as a barrier in generic primacy care implementation research [35–37], suggesting that limited time is an endemic challenge in primary care settings. Our findings from the stepped care context reinforce these observations and demonstrate that time should be recognized as a distinct and influential factor when planning and supporting implementation in primary care.

Among facilitators, resources emerged as the most prominent. Sufficient resources, such as staffing, funding, and infrastructure, were found to smooth implementation. The dual role of resources, acting as both a barrier when lacking and a facilitator when present, reflects findings from other studies on stepped care implementation [17]. However, resources are rarely emphasized as facilitators in the literature on stepped care implementation, where they are typically framed as barriers [18, 19, 21]. This dual function therefore appears to be a second novel finding of this study. Information and communication were also identified as key facilitators, consistent with prior research on stepped care implementation [17, 20], and generic primary-care implementation research [36].

The second research question posed: “How do these barriers and facilitators differ across aggregated, organization, and profession levels?” To address this, we examined barriers and facilitators at three levels: (1) an aggregated level encompassing all professions and organizations, (2) the organizational level, and (3) the professional level.

Existing research on complex interventions like stepped care has predominantly focused on barriers and facilitators at an aggregated level across regions, districts, or multiple organizations [17–19, 21]. While this approach provides a broad overview, it often obscures differences at the organizational or professional level. Our findings suggest that aggregated data, though useful for identifying general trends, may fail to capture the nuanced factors critical for implementation success in specific contexts. This represents a novel contribution, as stepped-care implementation studies have not previously compared levels of analysis or examined potential losses of information when relying solely on aggregated data.

Our study demonstrates that examining barriers and facilitators at the organizational level reveals unique, context-specific factors that are more relevant to individual organizations than aggregated data. Organizational readiness for implementation in generic implementation research, as emphasized in the CFIR [22], further underscores the importance of this level of analysis. The identification of the organizational level as the most informative and actionable level for understanding stepped-care implementation barriers and facilitators is a novel empirical finding in this field.

At the professional level, prior generic implementation research indicates that different professions encounter distinct barriers and facilitators due to variations in roles and norms [23]. However, our findings did not reveal clear profession-specific trends across organizations, suggesting that barriers and facilitators are not primarily linked to professional roles. This absence of distinct profession-level patterns contradicts expectations from generic implementation research, where individual health professional factors are given prominence [29], and constitutes also a new insight in stepped-care implementation. Nevertheless, existing generic implementation research suggests that involving all health professions in various phases of developing and implementing new models, such as the stepped care model, remains crucial for securing buy-in [24].

An exception was observed among psychologists, who demonstrated consensus on facilitators and barriers within two organizations. This consensus, however, was organization-specific, reinforcing the importance of organizational context over professional affiliation. The alignment among psychologists may stem from the theoretical foundations of stepped care, which often draw on psychological approaches such as cognitive behavioral therapy (CBT) [12]. Differences in theoretical orientations or different schools of psychotherapy, such as psychodynamic therapy (PDT), may further explain variations within this group [38]. The organization-specific alignment among psychologists, rather than a profession-wide pattern, provides a nuanced and previously unreported insight: organizational context for psychologists appears more influential than professional identity in shaping perceptions of stepped-care implementation.

The third research question was: “At which level should the barriers and facilitators be identified to develop effective implementation strategies?” Our findings indicate that the organizational level is the most effective for capturing barriers and facilitators. This conclusion reflects the observation that these factors are more closely tied to organizational context than to professions or aggregated data. Additionally, our study identified seven prominent barriers and facilitators alongside 17 additional factors across the three organizations, illustrating the diversity of individual experiences. Attempting to address this diversity at the individual level would be impractical and inefficient. Instead, focusing on the organizational level balances meaningful variation with practicality, enabling more actionable insights. This offers new empirical support for designing implementation strategies at the organizational level in stepped-care contexts, an area previously unexplored in the stepped care implementation literature.

The wide range of barriers and facilitators identified in this and prior studies on stepped care implementation [17–19, 21] reflects the inherent complexity of implementing stepped care. This complexity likely contributes to the moderate outcomes often observed in implementation efforts, such as the 4% to 12% behavior change reported in generic implementation studies [39]. Focusing on the organizational level to identify barriers and facilitators enables a more targeted understanding of key factors affecting implementation and supports the development of context-sensitive strategies.

Limitations.

This study provides important insights on the implementation of the stepped care model for mental health, but several limitations should be considered when interpreting the results. First, the reliance on self-reported data from healthcare professionals introduces the possibility of response bias. Participants may have answered in ways they perceived as socially acceptable or expected by the researchers, which could lead to inaccurate or skewed responses regarding barriers and facilitators. Second, the study’s sample consists of healthcare professionals from only three primary care organizations in Sweden, with a relatively small number of participants, which may not fully capture the diversity of healthcare systems, organizations, or professional roles. Consequently, the findings should be interpreted as exploratory. An exploratory study is appropriate for generating early insights but limits the extent to which firm conclusions can be drawn. Furthermore, because all data were collected from three organizations within a single Swedish primary care context, the generalizability of the findings is limited. Differences in organizational structures, healthcare systems, and cultural contexts in other countries may restrict the applicability of these results beyond the Swedish setting.

Third, despite efforts to increase participation, the response rate remains a limitation. The inclusion of offline paper surveys in some organizations suggests that some professionals may have been either unable or unwilling to complete the online survey, potentially leading to a less representative sample. Furthermore, GDPR regulations prevented us from obtaining exact staff numbers for each organization at the time of data collection. Although publicly available information enabled us to estimate the approximate staffing composition, these estimates allowed only an approximation of the overall response rate. The absence of precise staffing numbers for each professional group limits our ability to calculate profession-specific response rates. Consequently, the representativeness of some occupational groups, particularly smaller ones, cannot be fully assessed and should be considered when interpreting the findings.

Fourth, the study captures data at a single point in time, limiting the ability to track how barriers and facilitators evolve throughout the implementation process. As the stepped care model continues to be introduced and refined, barriers and facilitators may shift, and new challenges may emerge that were not captured during the survey period.

Fifth, the lack of consistent findings across most professions, with the partial exception of psychologists, should be interpreted with caution. The absence of clear profession-level patterns may reflect both the modest sample sizes within each professional group and potential limitations in the survey’s ability to capture profession-specific experiences. While our findings suggest that organizational context may be more influential than professional identity, it is also possible that more targeted or profession-sensitive items would have revealed additional differences. Future research should therefore consider using larger profession-specific samples and/or developing items tailored to the distinct roles, responsibilities, and clinical orientations of different health professions. It may also be valuable to adopt longitudinal designs and include a broader range of organizations to enhance the relevance and applicability of findings across healthcare systems.

These limitations should be considered when evaluating the findings and their relevance to broader implementation strategies.

## Conclusions

This exploratory survey study provides new insights into the early implementation of a stepped care model for mental health in Swedish primary care by identifying the barriers and facilitators that influence implementation across three organizations. While several findings align with previous research on stepped-care implementation and generic primary-care change processes, the study also offers three novel contributions that deepen understanding of how stepped care is adopted in real-world primary care settings.

First, although six of the seven prominent barriers and facilitators correspond to those previously reported in the literature, the study identifies “time required to work on implementation” as a distinct and previously unreported barrier in stepped-care research. While time may be considered part of general resource constraints, our findings show that it functions as a critical factor in its own right, warranting explicit consideration when planning and supporting implementation efforts.

Second, the study demonstrates that resources act not only as barriers when lacking but also as facilitators when present. This dual role has not been emphasized in earlier stepped-care implementation research, where resources are typically framed solely as barriers. Identifying resources as a facilitator therefore represents an additional novel contribution.

Third, by analyzing barriers and facilitators at aggregated, organizational, and professional levels, the study shows that the organizational level is the most informative and actionable level for identifying factors relevant to implementation success. Prior stepped-care implementation studies have not compared levels of analysis. Our findings indicate that aggregated data obscure important contextual nuances, while the individual level introduces a degree of diversity that would be impractical to address. The results therefore provide new empirical support for prioritizing the organizational level when designing implementation strategies for stepped-care models. The study also implicates that, contrary to expectations from generic implementation research, profession-specific patterns were largely absent, suggesting that organizational context plays a more influential role than professional identity. An exception was found among psychologists, who showed organization-specific, but not profession-wide, consensus, further underscoring the primacy of organizational context.

While the study offers valuable insights, its limitations, including response bias, the limited sample size, and the snapshot nature of the data, should be considered. Future research could build on these findings by exploring how barriers and facilitators evolve over time and by incorporating a broader range of organizations and professionals to enhance the generalizability of the results.

## Supplementary Information

Additional File 1.

Additional File 2.

Additional File 3.

## Data Availability

All data generated or analysed during this study are included in this published article and its supplementary information files.

## Abbreviations

CFIR: Consolidated Framework for Implementation Research
CBT: Cognitive behavioral therapy
PDT: Psychodynamic therapy
