# Supplementary material for: Why aggregated data falls short: an exploratory survey study on barriers and facilitators in implementing the stepped care model for mental health in primary care
Source: BMC Prim Care. 2026 Feb 28;27:120. doi: 10.1186/s12875-026-03238-0 (PMC13064063; doi:10.1186/s12875-026-03238-0)
Supplement: Supplementary file 1 — Additional File 1. [file 12875_2026_3238_MOESM1_ESM.docx]

**Additional file 1**

**Estimating the number of staff in the three primary care organizations**

In Sweden, national guidance from the National Board of Health and Welfare recommends a target ratio of one specialist in general practice per 1,100 inhabitants [1]. However, empirical workforce data indicate that actual staffing levels diverge substantially from this benchmark. A nationwide staffing survey conducted by the Swedish Medical Association in 2025 reported that a full-time specialist in general practice is, on average, responsible for approximately 1,750 listed patients in primary care [2].

For other professional groups, national data are considerably more limited. In practice, observational data from Swedish regional healthcare organizations and follow-up reports suggest that a medium-sized primary care organization (≈7,000–9,000 listed patients) typically employs approximately 4–6 general practitioners (plus trainees), around 6–10 full-time nurses or district nurses, 1–2 psychologists or psychotherapists, and roughly 0.5–1.5 full-time counsellors [3,4].

Based on these established staffing ranges, we applied the mean estimated value within each category to an organization serving approximately 8,000 listed patients. This yields the following approximate staffing levels:

Staffing ratios per 8,000 listed patients:
– Physicians: 5.3 ≈ 5
– Nurses: 8
– Psychologists: 1.6 ≈ 2
– Counselors: 0.8 ≈ 1

Applied to each organization:

| Organization | Listed patients | Medical doctors | Nurses | Psychologists | Counselors |
| --- | --- | --- | --- | --- | --- |
| 1 | 19,200 | 12.7 ≈ 13 | 19.2 ≈ 19 | 3.8 ≈ 4 | 1.9 ≈ 2 |
| 2 | 13,000 | 8.6 ≈ 9 | 13.0 ≈ 13 | 2.4 ≈ 2 | 1.3 ≈ 1 |
| 3 | 7,200 | 4.8 ≈ 5 | 7.2 ≈ 7 | 1.4 ≈ 1 | 1.0 ≈ 1 |

Subtotal:
Medical doctors =  27
Nurses =  39
Psychologists =  9
Counselors =  4
**Total = 79**

These derived values represent approximations based on national staffing norms and observed patterns in Swedish primary care, rather than actual headcounts at the included organizations.

**Reference list**

1. National Board of Health and Welfare (Socialstyrelsen). *Nationellt kunskapsstöd för god bemanning i primärvården: riktvärden för allmänmedicin*. Stockholm: Socialstyrelsen; 2018. URL: [https://www.socialstyrelsen.se/kunskapsstod-och-regler/omraden/god-och-nara-vard/fast-lakarkontakt/](https://www.socialstyrelsen.se/kunskapsstod-och-regler/omraden/god-och-nara-vard/fast-lakarkontakt/?utm_source=chatgpt.com) (last accessed 2025-11-24)
2. Swedish Medical Association (Läkarförbundet). *Bemanningsenkäten 2025: arbetsbelastning och listningstryck i svensk primärvård*. Stockholm: Sveriges läkarförbund; 2025. URL: [https://slf.se/app/uploads/2025/10/vardcentralernas-bemanning-rapport-lakarforbundet-pdf-1.pdf](https://slf.se/app/uploads/2025/10/vardcentralernas-bemanning-rapport-lakarforbundet-pdf-1.pdf?utm_source=chatgpt.com) (last accessed 2025-11-24)
3. Vårdgivare Halland. *Primärvårdens uppföljningsrapport – listning, tillgänglighet och bemanning*. Halmstad: Region Halland; 2021. URL: [https://www.regionhalland.se/download/18.6d31913f192bd56af4e13cd6/1729775598051/Region%20Hallands%20uppföljningsrapport%202%20januari%20-%20augusti%202024.pdf](https://www.regionhalland.se/download/18.6d31913f192bd56af4e13cd6/1729775598051/Region%20Hallands%20uppf%C3%B6ljningsrapport%202%20januari%20-%20augusti%202024.pdf?utm_source=chatgpt.com) (last accessed 2025-11-24)
4. Swedish Association of Local Authorities and Regions (SKR). *Regional workforce reports for primary care 2019–2023*. Stockholm: SKR; 2023. URL: [https://skr.se/download/18.14fb7b721997a8f53f23d1c7/1758791405604/Statistik-om-halso-och-sjukvard-samt-regional-utveckling-2023.xlsx](https://skr.se/download/18.14fb7b721997a8f53f23d1c7/1758791405604/Statistik-om-halso-och-sjukvard-samt-regional-utveckling-2023.xlsx?utm_source=chatgpt.com) (last accessed 2025-11-24)
