# Supplementary material for: Why aggregated data falls short: an exploratory survey study on barriers and facilitators in implementing the stepped care model for mental health in primary care
Source: BMC Prim Care. 2026 Feb 28;27:120. doi: 10.1186/s12875-026-03238-0 (PMC13064063; doi:10.1186/s12875-026-03238-0)
Supplement: Supplementary file 2 — Additional File 2. [file 12875_2026_3238_MOESM2_ESM.docx]

**Additional file 2. English translation of the survey**

**Research on the implementation of a stepped care model**

This survey is part of an ongoing study aimed at investigating how the implementation of stepped care can be carried out. The study is conducted in collaboration between (names anonymized). As part of this study, interviews have been conducted with staff at your healthcare center, and this survey serves as a continuation of that process.

We kindly ask you to respond regardless of whether you have participated in such an interview or not, in order to gain an overall picture of how all staff at your healthcare center perceive the implementation. The results will be presented in a way that ensures that no individuals or healthcare centers can be identified. Your answers are important, regardless of whether you have started working according to the stepped care model or not.

**Questions**

1. Which healthcare center do you work at?
   *(Response options: a list of three healthcare centers (anonymized) and an option for "Other, which?")*
2. What is your profession?
   *(Response options: nurse, district nurse, psychiatric nurse, psychologist, physician, physiotherapist, counselor, other; which?)*
3. Do you hold a managerial position (such as operations manager or team leader)?
   *(Response options: Yes or No)*
4. How many years have you worked in your professional role?
   *(Response options: open response)*
5. How many years have you worked at this healthcare center?
   *(Response options: open response)*
6. What is your gender?
   *(Response options: woman, man, other; which?)*
7. How much knowledge do you have about the stepped care model?
   *(Response options: no knowledge, very little knowledge, little knowledge, a lot of knowledge, very much knowledge)*
8. How does the stepped care model affect your work?
   *(Response options: to an extremely small extent, to a very small extent, to a small extent, to a moderate extent, to a high extent, to a very high extent, to an extremely high extent, don’t know)*
9. How much do you currently use the stepped care model in your work?
   *(Response options: to an extremely small extent, to a very small extent, to a small extent, to a moderate extent, to a high extent, to a very high extent, to an extremely high extent, don’t know)*

If you find it difficult to answer questions 10 and 11 below, you may find inspiration in the examples presented after question 11. These examples come from previously conducted interviews and research on introducing new work methods.

1. What are the most important facilitating factors for successfully implementing the stepped care model? Please list up to five factors and provide complete sentences.
2. What are the most important hindering factors for successfully implementing the stepped care model? Please list up to five factors and provide complete sentences.

**Inspiration for considering hindering or facilitating factors in the implementation of a stepped care model at our healthcare center:**

- The fundamental idea of the model is good.
- There is a lack of a shared vision.
- The model is flexible (adaptable).
- The benefits of the model are unclear.
- Management is involved in the implementation.
- Existing staff are not interested.
- Poor communication with staff.
- There is time to learn and participate in the implementation.
- It is possible to influence the implementation.
- It is not possible to influence the implementation.
- There is not enough available training.

Thank you for your participation! If you have any questions about the survey or wish to provide feedback, please feel free to contact the researchers at (university anonymized): (researchers anonymized).
