# Supplementary material for: Why aggregated data falls short: an exploratory survey study on barriers and facilitators in implementing the stepped care model for mental health in primary care
Source: BMC Prim Care. 2026 Feb 28;27:120. doi: 10.1186/s12875-026-03238-0 (PMC13064063; doi:10.1186/s12875-026-03238-0)
Supplement: Supplementary file 3 — Additional File 3. [file 12875_2026_3238_MOESM3_ESM.docx]

| **Additional file 3.** Factors influencing implementation of the stepped care model for mental health | | | | |
| --- | --- | --- | --- | --- |
| **Factor (n)** | **Organization 1** | **Organization 2** | **Organization 3** | **Total** |
| 1. Benefits of the model (barrier) | 3 | 0 | 2 | 5 |
| 1. Benefits of the model (facilitator) | 4 | 0 | 3 | 7 |
| 2. Clarity of the model (barrier) | 0 | 0 | 1 | 1 |
| 2. Clarity of the model (facilitator) | 0 | 0 | 2 | 2 |
| 3.Consensus (barrier) | 2 | 0 | 0 | 2 |
| 3.Consensus (facilitator) | 2 | 0 | 1 | 3 |
| 4.Flexibility of the model (barrier) | 0 | 0 | 0 | 0 |
| 4.Flexibility of the model (facilitator) | 0 | 0 | 3 | 3 |
| 5.Implementation guidelines (barrier) | 0 | 0 | 0 | 0 |
| 5.Implementation guidelines (facilitator) | 0 | 0 | 3 | 3 |
| 6.Influencing opportunities for staff (barrier) | 0 | 0 | 1 | 1 |
| 6.Influencing opportunities for staff (facilitator) | 4 | 0 | 1 | 5 |
| 7.Information and communication (barrier) | 3 | 0 | 3 | 6 |
| 7.Information and communication (facilitator) | 7 | 1 | 2 | 10 |
| 8.Interest (barrier) | 1 | 1 | 0 | 2 |
| 8.Interest (facilitator) | 0 | 1 | 0 | 1 |
| 9.Involvement of the entire organization (barrier) | 2 | 0 | 0 | 2 |
| 9.Involvement of the entire organization (facilitator) | 1 | 3 | 0 | 4 |
| 10.Knowledge of the model (barrier) | 2 | 1 | 1 | 4 |
| 10.Knowledge of the model (facilitator) | 1 | 1 | 1 | 3 |
| 11.Other (barrier) | 3 | 2 | 1 | 6 |
| 11.Other (facilitator) | 2 | 1 | 3 | 6 |
| 12.Parallel projects (barrier) | 1 | 1 | 0 | 2 |
| 12.Parallel projects (facilitator) | 0 | 0 | 0 | 0 |
| 13.Patient needs (barrier) | 1 | 0 | 0 | 1 |
| 13.Patient needs (facilitator) | 2 | 1 | 0 | 3 |
| 14.Propensity to change (barrier) | 2 | 3 | 1 | 6 |
| 14.Propensity to change (facilitator) | 1 | 0 | 0 | 1 |
| 15.Resources (barrier) | 6 | 2 | 2 | 10 |
| 15.Resources (facilitator) | 6 | 2 | 1 | 9 |
| 16.Routines (barrier) | 0 | 1 | 1 | 2 |
| 16.Routines (facilitator) | 0 | 0 | 0 | 0 |
| 17.Simplicity of the model (barrier) | 0 | 0 | 0 | 0 |
| 17.Simplicity of the model (facilitator) | 1 | 1 | 2 | 4 |
| 18.Staffing (barrier) | 0 | 0 | 2 | 2 |
| 18.Staffing (facilitator) | 0 | 0 | 1 | 1 |
| 19.Stress (barrier) | 0 | 1 | 2 | 3 |
| 19.Stress (facilitator) | 0 | 0 | 0 | 0 |
| 20.Support from management (barrier) | 1 | 0 | 2 | 3 |
| 20.Support from management (facilitator) | 2 | 2 | 3 | 7 |
| 21.Time to work with implementation (barrier) | 5 | 2 | 6 | 13 |
| 22.Time to work with implementation (facilitator) | 4 | 1 | 3 | 8 |
| 23.Training needed (barrier) | 1 | 0 | 1 | 2 |
| 23.Training needed (facilitator) | 2 | 0 | 2 | 4 |
| 24.Workload with the model (barrier) | 3 | 0 | 2 | 5 |
| 24.Workload with the model (facilitator) | 1 | 0 | 0 | 1 |
